# Dual-Function Nitrogen Modification of Phenolic Resin Pyrolytic Carbon: A g-C_3_N_4_ Protective Phase and Skeletal Nitrogen Doping for Enhanced Oxidation Resistance

**DOI:** 10.3390/ma19173585

**Published:** 2026-08-24

**Authors:** Pengcheng Jiang, Huidong Tang, Xin Xiong, Wenting Wang, Kang Long, Zhiwen Li, Yongming Kang, Xinwei Ou, Zhi Wu

**Affiliations:** School of Materials Science and Engineering, Hunan Institute of Technology, Hengyang 421002, China; tanghuidong@hnit.edu.cn (H.T.); wangwtwx@163.com (W.W.); a200508192021@163.com (Z.L.);

**Keywords:** phenolic resin, nitrogen modification, melamine, graphitic carbon nitride, oxidation resistance

## Abstract

Phenolic resin pyrolytic carbon is a key matrix phase in carbon-based refractories and carbon/carbon composites; however, its defect-rich glassy carbon structure exhibits poor oxidation resistance at elevated temperatures. In this work, we report a facile one-step thermal-treatment strategy using melamine as a nitrogen source to prepare nitrogen-modified phenolic resin pyrolytic carbon (NC). The structural evolution and oxidation behavior of samples carbonized at 500–800 °C were systematically investigated by XRD, SEM, TEM, FT-IR, Raman, XPS, BET, and TG-DSC. The results reveal that melamine-derived nitrogen exists in two distinct forms: at 500–700 °C, a carbon nitride-rich phase consistent with graphitic carbon nitride (g-C_3_N_4_) forms sheet- and belt-like structures on the carbon surface and partially fills the internal pores; at 800 °C, its long-range crystalline signature disappears, while pyridinic, pyrrolic, and graphitic nitrogen remain in the carbon framework. From 500 to 800 °C, the relative N 1s fraction of pyridinic N decreases from 72.33% to 44.38%, whereas graphitic N increases from 0.47% to 24.09%. Meanwhile, the pore structure evolves from a mesopore-dominated architecture with a limited accessible surface area at 500–600 °C to a micropore-rich structure at 700–800 °C. Relative to unmodified PR-800, NC-800 exhibits an approximately 30 °C higher onset oxidation temperature and an approximately 40 °C higher complete oxidation temperature, together with a lower maximum mass-loss rate and a delayed, broadened exothermic response. These results show that melamine-derived pore regulation and skeletal nitrogen doping jointly retard oxygen transport and suppress oxidation-active defect sites, providing a simple and potentially scalable route for improving the high-temperature oxidation resistance of phenolic resin pyrolytic carbon.

## 1. Introduction

Phenolic resins are widely used as carbonaceous binders and matrix precursors in carbon-based refractories, carbon/carbon composites, and related high-temperature carbon materials because they combine suitable viscosity and wettability with a relatively high carbon yield after curing and carbonization [1,2,3]. However, phenolic resin pyrolytic carbons retain an isotropic, defect-rich glassy-carbon structure that is difficult to graphitize, even at elevated temperatures, which limits structural robustness [4,5,6]. More importantly, pyrolysis inevitably generates vacancies, dangling bonds, and other structural defects in the carbon framework. These defects act as preferential reaction sites for oxygen, initiating oxidation at relatively low temperatures and accelerating material degradation [7,8,9]. In practical applications such as refractories for continuous steel casting [10] and carbon/carbon composites for aerospace thermal-protection systems [11], premature oxidation of the phenolic resin pyrolytic carbon phase is a major cause of performance deterioration. Improving the oxidation resistance of phenolic-resin pyrolytic carbon is therefore important for extending component service life and reliability in oxidative high-temperature environments.

The final structure and properties of pyrolytic carbon depend on multiple factors, among which precursor chemistry plays a particularly decisive role. For example, Tkachenko et al. [12] employed sulfate lignin as the carbon precursor with urea and HNO_3_ during a one-step pyrolysis process at 800 °C, yielding a predominantly microporous structure with a specific surface area of approximately 1000 m^2^/g. Among various carbon precursors, phenolic resin stands out for its processing simplicity and high char yield, whereas the incorporation of heteroatom-containing modifiers provides an additional route to tune local bonding, defect chemistry, and pore development. Accordingly, heteroatom modification has emerged as an effective strategy for regulating the structure and properties of phenolic resin pyrolytic carbons, and modifiers containing nitrogen [13], boron [14], and phosphorus [15] have been widely investigated. Nitrogen modification is particularly attractive because nitrogen can be incorporated into aromatic carbon domains with limited lattice distortion and can occur as pyridinic, pyrrolic, and graphitic configurations [16]. These configurations alter the local electron density and chemical reactivity of neighboring carbon atoms. Recent studies have demonstrated that the nitrogen source and synthesis route can substantially affect the nitrogen configuration, pore structure, and functional properties of phenolic resin pyrolytic carbons. For example Xu et al. [17] synthesized nitrogen-doped phenolic resin pyrolytic carbon nanospheres via the Stöber method using melamine as the nitrogen source. The resulting nitrogen-doped carbon exhibited excellent performance toward peroxymonosulfate activation for bisphenol A (BPA) degradation, demonstrating the important role of nitrogen incorporation in regulating the surface chemical reactivity of phenolic resin pyrolytic carbon. Singh et al. [18] synthesized nitrogen-doped mesoporous carbon nanospheres using urea–phenol–formaldehyde resin as the precursor and F127 as a soft template via an aqueous route. The optimized M0.1 sample, with a nitrogen content of 1.52% and ordered mesopores, exhibited an exceptional CO_2_ adsorption capacity of 2.53 mmol g^−1^ at a 10% CO_2_ feed, owing to the synergistic effects of nitrogen functionalities (including pyrrolic, pyridine, and amine groups), ordered mesopores, and abundant micropores. Ma et al. [19] developed N/O/B co-doped phenolic resin pyrolytic porous carbon materials via a sol–gel method combined with high-temperature carbonization and KOH activation. The optimized sample, featuring a high specific surface area of 1721 m^2^ g^−1^, a hierarchical micro/mesoporous structure, and uniform N/O/B doping (N: 8.31 at.%; O: 14.11 at.%; B: 0.55 at.%), exhibited a superior specific capacitance of 377 F g^−1^ in a three-electrode system with excellent rate performance and long-term cycling stability. These studies demonstrate that precursor selection, nitrogen source, heteroatom configuration, and pore architecture can collectively regulate the physicochemical properties of phenolic resin pyrolytic carbons. Nevertheless, most previous studies have focused primarily on adsorption, electrochemical, catalytic, or flame-retardant applications, whereas their behavior under high-temperature oxidative conditions has received comparatively limited attention. In particular, the coupled relationships among nitrogen configuration, carbon-nitride phase evolution, pore accessibility, and oxidation resistance remain insufficiently understood.

The choice of nitrogen precursor plays a critical role in determining the nitrogen content, configuration, and overall structural evolution of the resulting nitrogen-modified carbons [20]. Commonly used nitrogen sources include melamine, urea, dicyandiamide, and acrylamide, each of which exhibits distinct decomposition behaviors and nitrogen retention efficiencies during carbonization. For example, Chen et al. [21] systematically compared melamine, urea, dicyandiamide, and acrylamide as nitrogen sources for biomass-derived porous carbon and found that melamine yielded the highest surface nitrogen content (4.54%) and the highest pyrrolic nitrogen proportion (53.19%), significantly higher than those obtained using urea (39.06%) and dicyandiamide (47.17%). The superior nitrogen retention and pyrrolic-N enrichment achieved with melamine were attributed to enhanced surface wettability, improved polarization, and stronger K^+^ adsorption, as confirmed by DFT calculations, ultimately leading to the highest specific capacitance (349.3 F g^−1^). These findings demonstrate that the chemical nature and thermal evolution of the nitrogen precursor can strongly influence both nitrogen retention and the resulting carbon structure. In this context, melamine is particularly attractive for phenolic resin modification. Melamine (C_3_H_6_N_6_) possesses a high nitrogen content (66.7 wt%) and a rigid triazine-ring structure (1,3,5-triazine-2,4,6-triamine) [22]. The triazine rings can facilitate C–N bond formation and promote the development of graphitic-like layered structures during thermal condensation [23]. More importantly, melamine can undergo thermal condensation via deamination and polycondensation at temperatures above 300 °C, with the formation of intermediate condensed species such as melem, followed by further polymerization toward g-C_3_N_4_ at approximately 500–550 °C [24]. g-C_3_N_4_ is a two-dimensional layered polymeric semiconductor composed solely of carbon and nitrogen, with a graphene-like layered architecture [25]. Its layered morphology, nitrogen-rich framework, and ability to form in situ during thermal treatment make g-C_3_N_4_ particularly relevant to the structural regulation of carbon matrices. When melamine is introduced into phenolic resin, the in situ formation of g-C_3_N_4_ during carbonization may provide a physical barrier layer that hinders oxygen diffusion, while nitrogen-containing species generated during the thermal transformation and partial decomposition of the carbon-nitride phase may simultaneously become incorporated into the carbon framework. This dual functionality—combining a protective g-C_3_N_4_ phase with chemically incorporated nitrogen—offers a distinctive pathway to simultaneously regulate pore architecture and passivate oxidation-active sites in phenolic resin pyrolytic carbons. Accordingly, melamine provides a rational precursor for constructing a dual nitrogen-modification architecture in which phase-level protection and atomic-scale nitrogen doping may act cooperatively to enhance oxidation resistance.

In this work, a facile one-step thermal-treatment method was used to prepare nitrogen-modified phenolic resin pyrolytic carbon (NC) using melamine as both the nitrogen source and the g-C_3_N_4_ precursor. The structural evolution, nitrogen configuration transformation, pore development, and oxidation behavior of the resulting NC samples are systematically investigated by XRD, SEM, TEM, FT-IR, Raman, XPS, BET, and TG-DSC analysis. The existence forms of nitrogen in the carbon matrix are systematically characterized, and the evolution of the pore structure and oxidation behavior are systematically characterized. The novelty of this work lies in the systematic elucidation of the dual-function mechanism of melamine-derived nitrogen—combining a protective g-C_3_N_4_ phase with chemically incorporated nitrogen configurations—and its correlation with the structural evolution and oxidation resistance of phenolic resin pyrolytic carbons. This work not only fills the knowledge gap regarding the role of melamine-derived nitrogen in structural regulation and oxidation protection but also provides a simple and potentially scalable strategy for extending the high-temperature service life of phenolic-resin-based carbon materials in oxidative environments.

## 2. Experimental Section

### 2.1. Materials

Liquid thermosetting phenolic resin (PR, Jiesheng Chemical Co., Ltd., Wuhan, China) was used as the carbon precursor. Melamine (C_3_H_6_N_6_, >99.0%, Sinopharm Chemical Reagent Co., Ltd., Beijing, China) served as the nitrogen source. Ethanol (analytical grade, Sinopharm Chemical Reagent Co., Ltd., Beijing, China) was used as the solvent.

### 2.2. Preparation of Nitrogen-Modified Phenolic Resin Pyrolytic Carbon

**Figure 1:**
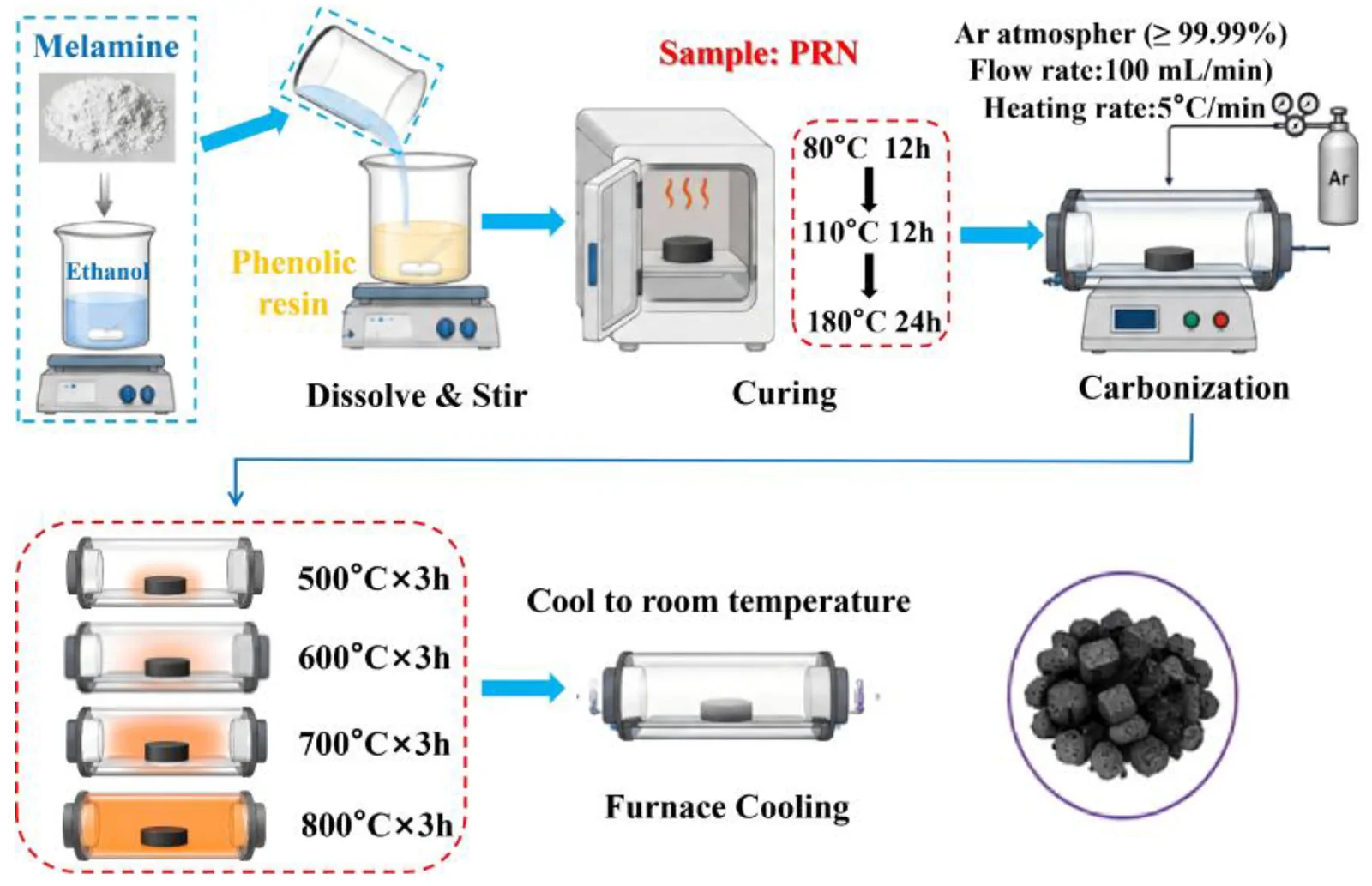
Schematic diagram of the preparation of nitrogen-modified phenolic resin pyrolytic carbons via melamine-assisted thermal treatment.

Melamine powder was added to ethanol and magnetically stirred to form a homogeneous dispersion. The melamine-containing dispersion was then slowly introduced into the phenolic resin under continuous stirring to ensure uniform mixing. The mass ratio of melamine, ethanol, and phenolic resin was fixed at 1:1:4. The mixture was cured successively at 80 °C for 12 h, 110 °C for 12 h, and 180 °C for 24 h, and the cured precursor was designated PRN. PRN was subsequently placed in a tube furnace and carbonized under flowing argon (purity ≥ 99.99%, flow rate 100 mL/min) at a heating rate of 5 °C/min. Independent specimens were heated to target temperatures of 500, 600, 700, and 800 °C; held for 3 h; and then furnace-cooled to room temperature under argon. The resulting nitrogen-modified phenolic resin pyrolytic carbons were denoted NC-500, NC-600, NC-700, and NC-800, respectively. The preparation route is schematically illustrated in Figure 1. For comparison, unmodified phenolic resin (PR) was cured under identical conditions and independently carbonized at 500, 600, 700, and 800 °C using the same heating and holding protocol. The resulting reference samples were designated PR-500, PR-600, PR-700, and PR-800, respectively.

### 2.3. Characterization

The crystalline phases of the samples were analyzed by X-ray diffraction (XRD, X’Pert Pro MPD, Philips, Eindhoven, The Netherlands) using Cu Kα radiation (λ=0.15406 nm) over the 2θ range of 10–90°. Powder samples were evenly pressed onto a flat glass holder for measurement. The morphology and microstructure were examined by field-emission scanning electron microscopy (FESEM, Nova400 NanoSEM, FEI, Hillsboro, OR, USA) and high-resolution transmission electron microscopy (HRTEM, JEM-2100UHR, JEOL, Tokyo, Japan), both equipped with energy-dispersive X-ray spectroscopy (EDS). For SEM observation, powder samples were dispersed on carbon tape and sputter-coated with a thin gold layer to enhance conductivity. For TEM observation, samples were ultrasonically dispersed in ethanol, dropped onto carbon-coated copper grids, and dried at room temperature before examination.

Fourier-transform infrared spectra (FT-IR, Nicolet Avatar FT-IR 360, Thermo Fisher Scientific, Waltham, MA, USA) were recorded in the range of 400–4000 cm^−1^ using the KBr pellet technique with a spectral resolution of 4 cm^−1^ and 32 scans per spectrum. The samples were mixed with KBr at a mass ratio of approximately 1:100 and pressed into transparent pellets. All spectra were baseline-corrected and normalized for comparison. The structural evolution of the carbon framework was evaluated by a laser confocal micro-Raman spectrometer (Raman, inVia Qontor, Renishaw, Wotton-under-Edge, UK). All spectra were recorded at room temperature using a 532 nm excitation laser with a grating of 1800 lines/mm. The intensity ratio I_D_/I_G_ was determined from the integrated areas of the D band (~1350 cm^−1^), associated with structural defects and disorder, and the G band (~1580 cm^−1^), corresponding to the E_2_g vibrational mode of sp^2^-bonded carbon atoms. The surface elemental composition and nitrogen chemical states were analyzed by X-ray photoelectron spectroscopy (XPS, Thermo ESCALAB250XI, Thermo Fisher Scientific, East Grinstead, West Sussex, UK) with Al Kα radiation (hν=1486.68 eV). The XPS spectra were acquired with a pass energy of 50 eV for survey scans and 20 eV for high-resolution scans. All binding energies were calibrated against the C 1s peak at 284.8 eV. Peak deconvolution of the N 1s high-resolution spectra was performed using XPSPEAK 4.1 software with a Shirley-type background subtraction and a mixed Gaussian–Lorentzian (70:30) peak shape. The full width at half maximum (FWHM) of each component was constrained within the range of 1.2–1.8 eV to ensure physically meaningful fitting results. The pore structures of the samples were characterized by N_2_ adsorption–desorption measurements using an automated gas-sorption analyzer (BET, Autosorb-1-MP, Quantachrome Instruments, Boynton Beach, FL, USA). Before measurement, the samples were degassed under vacuum at 200 °C for 3 h. The adsorption–desorption isotherms were recorded using nitrogen as the adsorbate at 77 K. The specific surface area was calculated using the Brunauer–Emmett–Teller (BET) method in the relative pressure (P/P_0_) range of 0.05–0.30. The pore-size distribution, cumulative pore-volume distribution, average pore diameter (D_p_), and total pore volume (V_t_) were determined from the desorption branch using the Barrett–Joyner–Halenda (BJH) method. The oxidation behaviors of the paired PR and NC samples carbonized at 500–800 °C were evaluated by thermogravimetric–differential scanning calorimetry (TG–DSC, STA 449C, Netzsch, Selb, Germany). The measurements were performed from room temperature to 1000 °C in air at a heating rate of 10 °C min^−1^. DTG curves were obtained from the first derivative of the TG data with respect to temperature.

## 3. Results and Discussion

### 3.1. Phase Composition and Microstructure of NC

**Figure 2:**
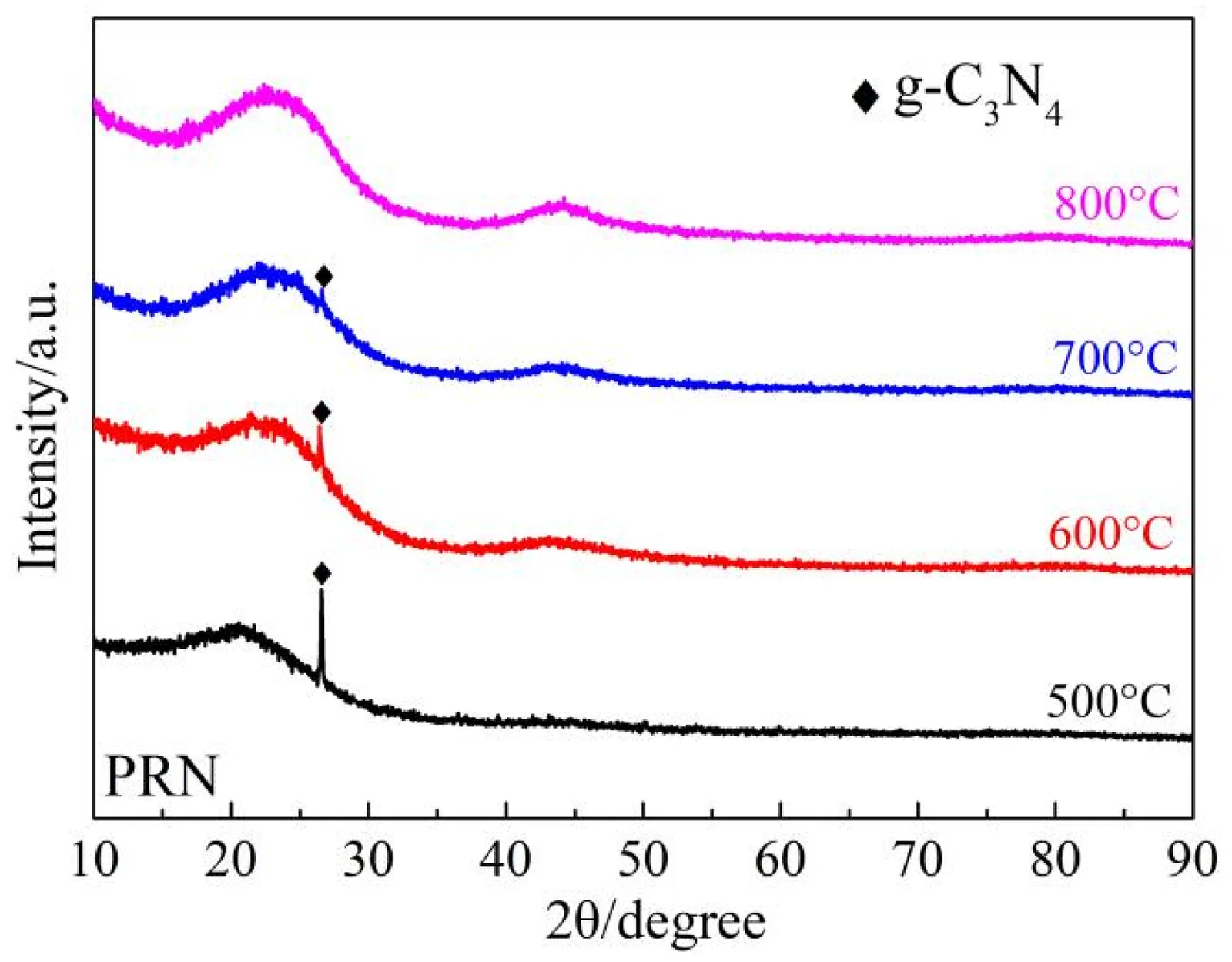
XRD patterns of samples NC obtained by calcining PRN at 500–800 °C.

XRD was used to examine the phase evolution of PRN carbonized at 500–800 °C and to determine whether a crystalline carbon-nitride phase formed within the phenolic resin pyrolytic carbon matrix. The XRD patterns of the NC samples obtained by calcining PRN at 500–800 °C are shown in Figure 2.

As shown in Figure 2, all samples exhibit broad diffraction features, indicating that the NC samples largely retain a disordered glassy-carbon structure after pyrolysis. For NC-500, NC-600, and NC-700, a diffraction peak appears at approximately 27.3°, which can be assigned to the (002) plane of g-C_3_N_4_ (JCPDS No. 87-1526) [26]. This result indicates that melamine can undergo thermal polymerization during the pyrolysis of phenolic resin, forming g-C_3_N_4_ that remains in the resin-derived carbon matrix. The intensity of the g-C_3_N_4_ diffraction peak gradually decreases with increasing calcination temperature from 500 to 700 °C, indicating that the g-C_3_N_4_ structure becomes progressively weakened or partially decomposed at elevated temperatures. When the calcination temperature increases to 800 °C, the characteristic diffraction peak of g-C_3_N_4_ is no longer clearly observed, suggesting that crystalline g-C_3_N_4_ is largely decomposed or transformed under this condition. Therefore, the XRD results demonstrate that g-C_3_N_4_ exists in the phenolic resin pyrolytic carbon matrix mainly within the temperature range of 500–700 °C, confirming that melamine derived g-C_3_N_4_ is one important form of nitrogen in the NC samples.

**Figure 3:**
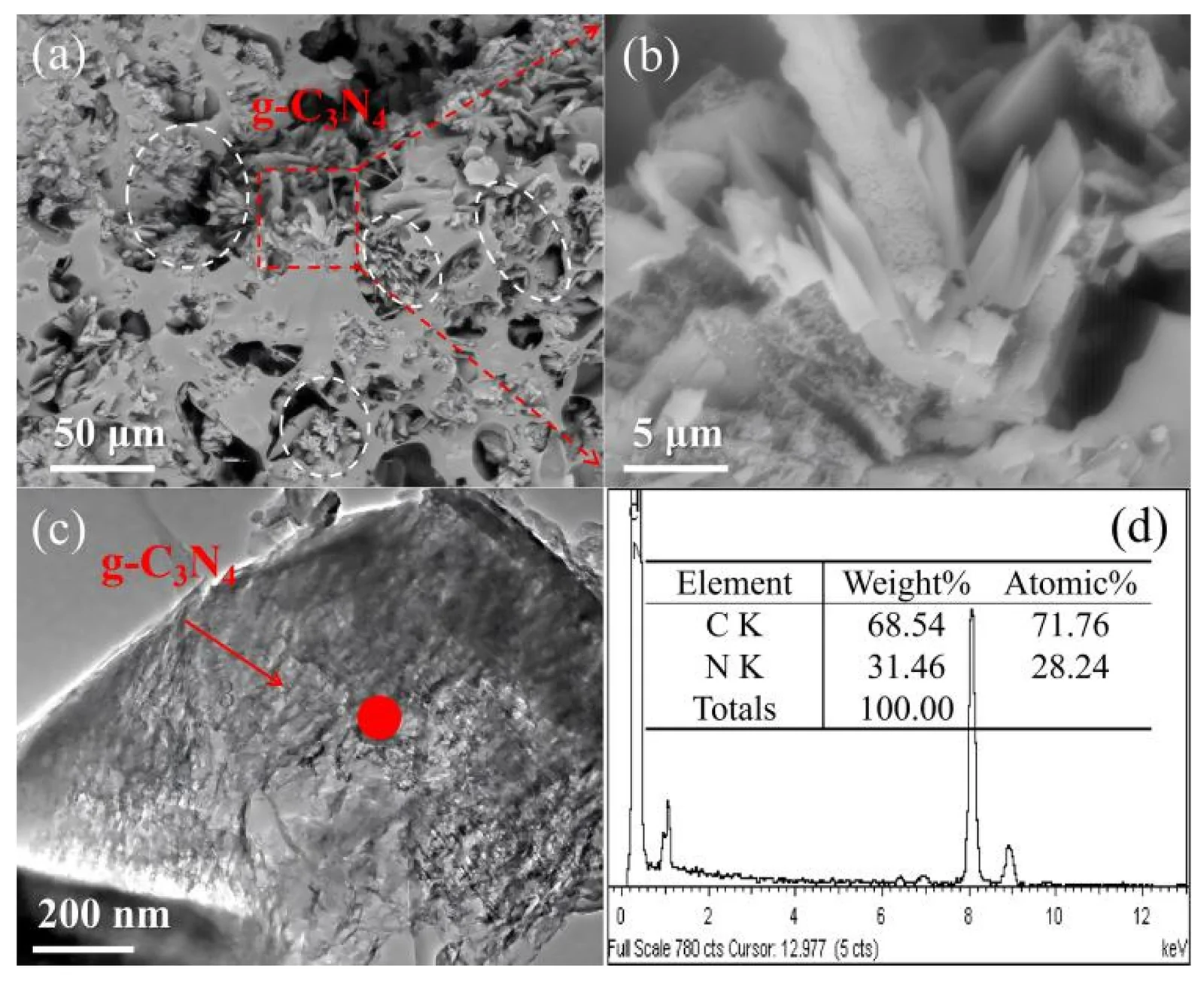
SEM and TEM images of the sample PRN calcined at 500 °C. (**a**,**b**) SEM images, (**c**) TEM image, and (**d**) EDS spectrum of the selected region in (**c**).

The XRD results confirmed the formation of g-C_3_N_4_ in the samples NC calcined at 500–700 °C. SEM and TEM observations were further performed on the NC sample calcined at 500–700 °C to clarify the microstructural features of melamine-derived g-C_3_N_4_ and its relationship with the porous resin-derived carbon matrix. Figure 3 shows the SEM and TEM images of the sample PRN calcined at 500 °C. As shown in Figure 3a, NC-500 exhibits a porous matrix formed during phenolic-resin pyrolysis. Numerous sheet-like structures are distributed on the carbon surface and within the pores. The enlarged SEM image in Figure 3b shows that these structures form lamellar stacks and partially occupy the pore space. The TEM image in Figure 3c further reveals a thin, stacked morphology. EDS analysis of the selected lamellar region in Figure 3d detects mainly C and N, with atomic fractions of 71.76% and 28.24%, respectively. Taken together with the XRD result, these observations support the assignment of the lamellar structures to a carbon-nitride-rich phase consistent with g-C_3_N_4_ [27]. EDS alone, however, does not uniquely identify the phase. The sheets appear to be attached to the carbon matrix and to partially occupy the pores. It is therefore reasonable to propose that they may restrict oxygen-transport pathways and potentially improve the oxidation resistance of the modified carbon.

**Figure 4:**
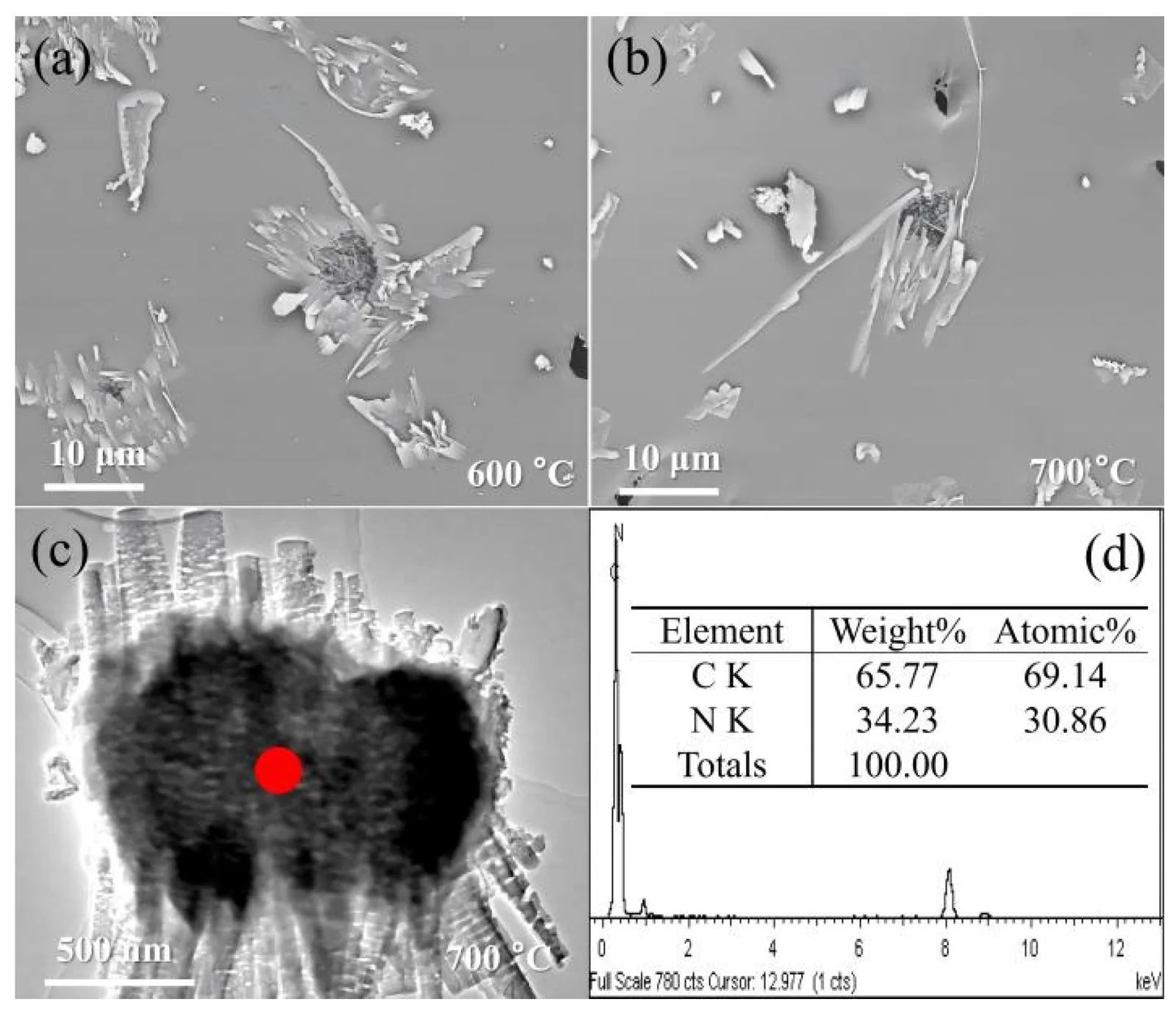
Morphological and elemental characterization of NC samples calcined at elevated temperatures: (**a**) SEM image of NC-600, (**b**) SEM image of NC-700, (**c**) TEM image of NC-700, and (**d**) EDS spectrum of the selected region in (**c**).

To further investigate the evolution of the melamine-derived carbon nitride phase with increasing heat-treatment temperature, the NC samples calcined at 600 and 700 °C were examined by SEM and TEM, as shown in Figure 4. As shown in Figure 4a, the NC-600 sample still contains a considerable number of elongated and sheet-like structures distributed on the resin-derived carbon matrix. When the calcination temperature is increased to 700 °C, the amount of these structures is noticeably reduced, as observed in Figure 4b, suggesting that part of the carbon nitride phase may decompose or transform at the higher temperature. Nevertheless, the remaining structures exhibit a more distinct layered morphology, with clearer lamellar stacking and more regular plate-like features. The TEM image in Figure 4c further reveals the layered structure of the residual phase in the NC-700 sample. The corresponding EDS analysis in Figure 4d shows that the selected region is mainly composed of C and N, with atomic contents of 69.14% and 30.86%, respectively, supporting its assignment to a carbon-nitride-rich phase. These observations indicate that, although the amount of sheet-like carbon nitride gradually decreases as the heat-treatment temperature increases from 600 °C to 700 °C, the layered characteristics of the remaining phase become more pronounced.

### 3.2. Evolution of Carbon Structure and Nitrogen Chemical States

**Figure 5:**
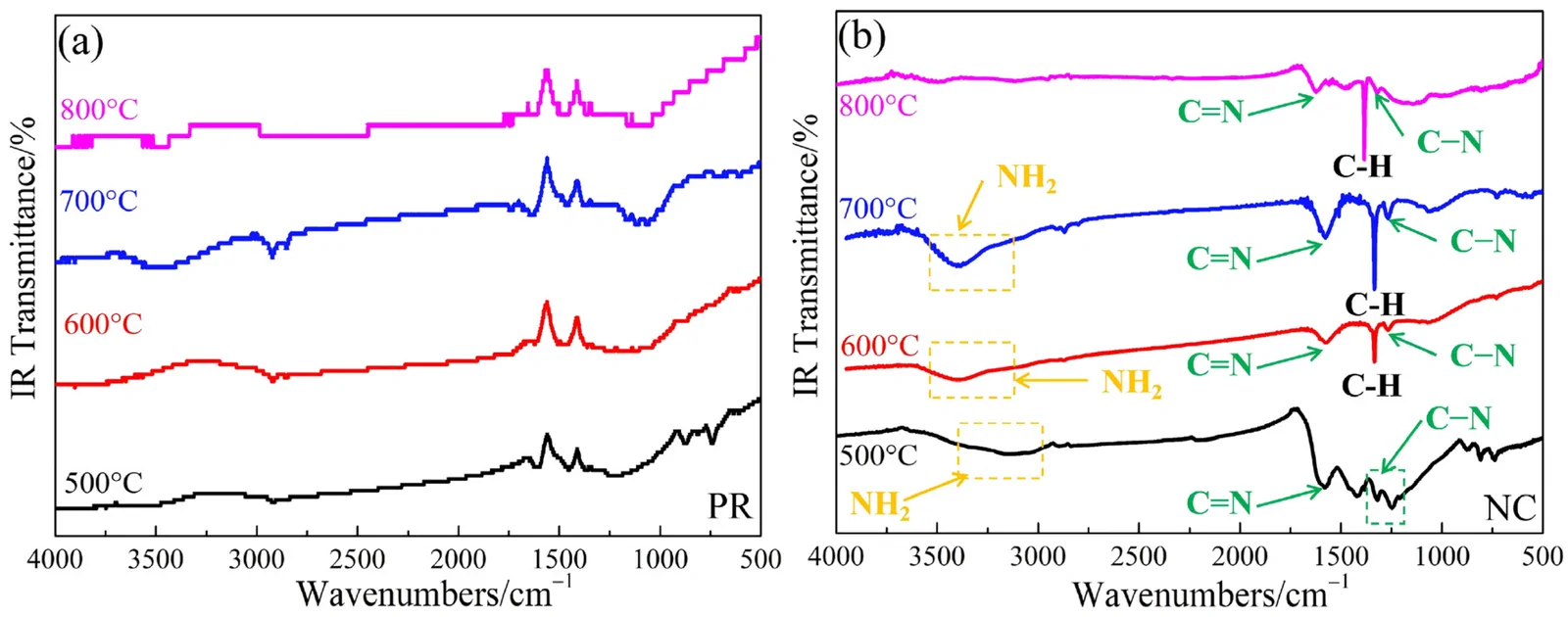
FT-IR spectra of PR and NC obtained at 500–800 °C: (**a**) PR, and (**b**) NC.

Although the amount of visible sheet-like g-C_3_N_4_ decreases at higher carbonization temperatures, nitrogen introduced by melamine may remain in chemically bound configurations within the carbonaceous framework. FT-IR, Raman and XPS analyses were therefore conducted to identify the nitrogen-containing bonds and to clarify the evolution of nitrogen chemical states in the NC samples. FT-IR analysis was conducted to identify the nitrogen-containing bonds and to clarify the evolution of nitrogen chemical states in the NC samples. Figure 5 presents the FT-IR spectra of both the unmodified PR samples (PR-500 to PR-800) and the NC samples (NC-500 to NC-800). All spectra were baseline-corrected and normalized for direct comparison.

The unmodified PR samples (Figure 5a) exhibit characteristic bands corresponding to the carbonized phenolic resin framework: C–H stretching vibrations at 2800–3000 cm^−1^, aromatic C=C stretching at approximately 1600 cm^−1^, and C–H out-of-plane bending at approximately 800 cm^−1^ [27]. No N–H- or C–N-related bands are observed in any PR sample, confirming the absence of nitrogen, as expected. The intensity of the C–H stretching band decreases with increasing carbonization temperature from 500 °C to 800 °C, consistent with the progressive dehydrogenation and aromatization of the carbon framework. The NC samples (Figure 5b) exhibit several additional bands compared with their PR counterparts. The broad absorption band in the range of approximately 3000–3600 cm^−1^ can be assigned to the stretching vibrations of –NH_2_ and/or –NH groups [28]. This band is relatively evident in NC-500, NC-600, and NC-700 but becomes significantly weakened at 800 °C, indicating the progressive removal or transformation of amino-containing species with increasing calcination temperature. The absorption band near 1600–1650 cm^−1^ is attributed to the stretching vibration of C=N bonds, while the bands located in the region of approximately 1200–1400 cm^−1^ are associated with C–N stretching vibrations [29]. Notably, these C=N- and C–N-related features are absent in the PR-800 spectrum, confirming that they originate from the melamine-derived nitrogen species. These features remain detectable throughout the investigated temperature range, indicating the persistence of nitrogen-containing chemical structures after carbonization. The feature near 808 cm^−1^ is commonly assigned to the out-of-plane breathing vibration of triazine or heptazine units, which is present in NC-500, NC-600, and NC-700 but becomes undetectable at 800 °C, consistent with the decomposition of g-C_3_N_4_ observed in the XRD results (Figure 2).

A notable observation from the normalized FT-IR spectra is that a C–H stretching band (2800–3000 cm^−1^) is clearly present in NC-600, NC-700, and NC-800 but is negligible in NC-500. The unmodified PR samples also exhibit C–H bands at all temperatures (Figure 5a), confirming that this feature is intrinsic to the carbonized resin framework. The absence of the C–H band in NC-500 can be attributed to two factors. First, at 500 °C, the phenolic resin is still in the early stage of carbonization, and the carbon framework has not yet undergone extensive dehydrogenation and aromatization. The relatively low degree of carbonization results in a broad and featureless FT-IR background, where the C–H signal is likely masked by the strong and broad N–H stretching vibration (3000–3600 cm^−1^) that is more pronounced at this temperature due to the abundant amino groups from melamine. Second, as the temperature increases to 600–800 °C, the phenolic resin undergoes progressive dehydrogenation and condensation reactions, leading to the formation of condensed aromatic structures with residual C–H bonds at the edges of graphitic domains [30]. The PR-800 spectrum shows a similar C–H band, further confirming that this feature is intrinsic to the carbonized resin framework and not related to nitrogen doping. The emergence of C–H vibrations in NC-600 to NC-800 therefore reflects the structural evolution from a cross-linked polymer network to a more condensed carbonaceous framework. The structural transformation of the carbon backbone will be further elucidated by Raman spectroscopy in the following section.

**Figure 6:**
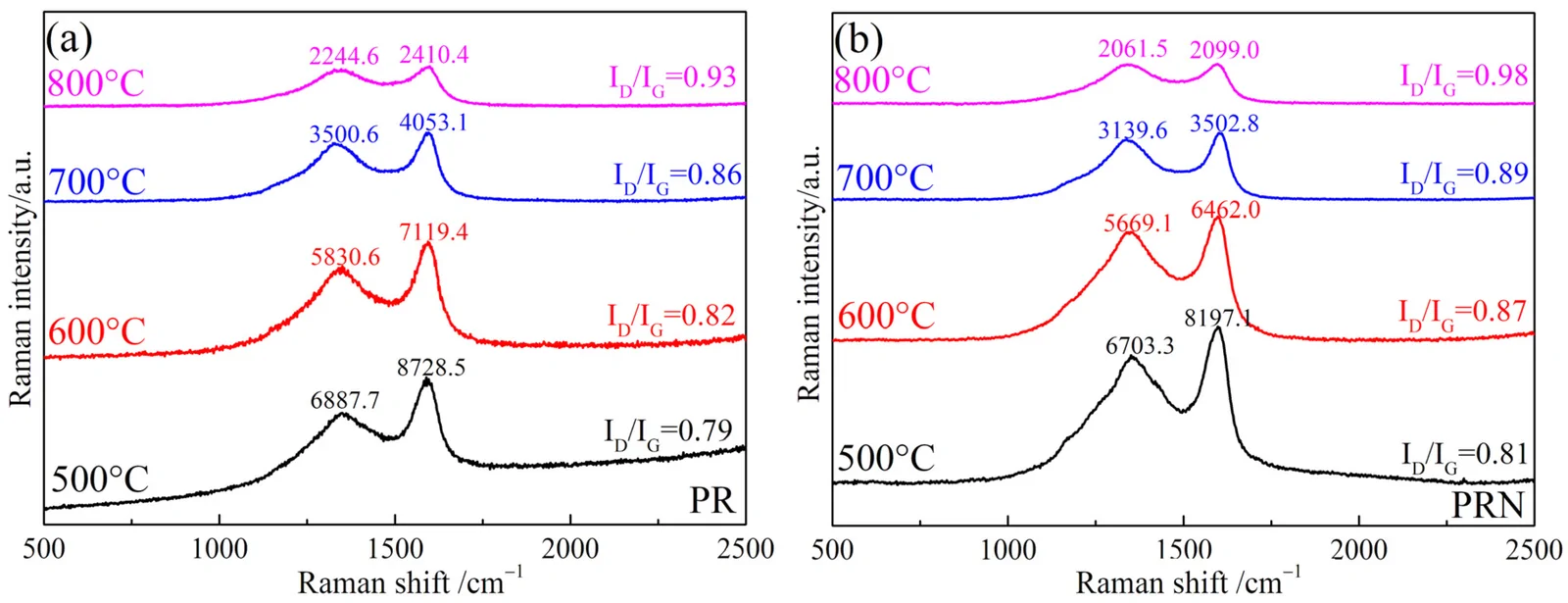
Raman spectra of PR and NC obtained at 500–800 °C: (**a**) PR and (**b**) NC.

To further clarify whether melamine-derived nitrogen modification alters the carbon backbone during pyrolysis, Raman spectroscopy was performed on both the unmodified PR and nitrogen-modified NC samples carbonized at 500–800 °C. Raman spectroscopy is particularly sensitive to the bonding configuration and defect structure of sp^2^ carbon and therefore provides complementary structural information to the FT-IR results. The corresponding Raman spectra are shown in Figure 6.

As shown in Figure 6, all PR and NC samples exhibit two characteristic bands located at approximately 1350 and 1580–1600 cm^−1^, corresponding to the D and G bands of carbonaceous materials, respectively. The D band is associated with defect-activated breathing vibrations of sp^2^-bonded carbon rings and is sensitive to structural defects, edge sites, and disruption of the aromatic carbon network, whereas the G band originates mainly from the in-plane stretching vibration of sp^2^-hybridized carbon atoms. The relative intensity ratio of the two bands (I_D_/I_G_) was therefore used to evaluate the evolution of the carbon framework. For the unmodified phenolic-resin pyrolytic carbons, the (I_D_/I_G_) values are 0.79, 0.82, 0.86, and 0.93 for PR-500, PR-600, PR-700, and PR-800, respectively. A similar temperature-dependent increase is observed for the nitrogen-modified samples, for which I_D_/I_G_ increases from 0.81 for NC-500 to 0.87, 0.89, and 0.98 for NC-600, NC-700, and NC-800, respectively. These results confirm that the carbon framework continuously evolves over the investigated carbonization temperature range. Because phenolic resin pyrolytic carbon remains predominantly a non-graphitic, glassy-carbon structure at 500–800 °C, the increase in I_D_/I_G_ is more appropriately associated with the rearrangement of aromatic sp^2^ domains and the evolution of defect and edge configurations rather than with conventional long-range graphitization. More importantly, at each carbonization temperature, the I_D_/I_G_ ratio of the nitrogen-modified sample is higher than that of the corresponding unmodified PR sample. Specifically, the ratios increase from 0.79 to 0.81 at 500 °C, from 0.82 to 0.87 at 600 °C, from 0.86 to 0.89 at 700 °C, and from 0.93 to 0.98 at 800 °C after nitrogen modification. This systematic difference demonstrates that melamine addition modifies the carbon backbone and produces a greater population of Raman-active defect/edge environments or local distortions within the sp^2^ carbon network.

**Figure 7:**
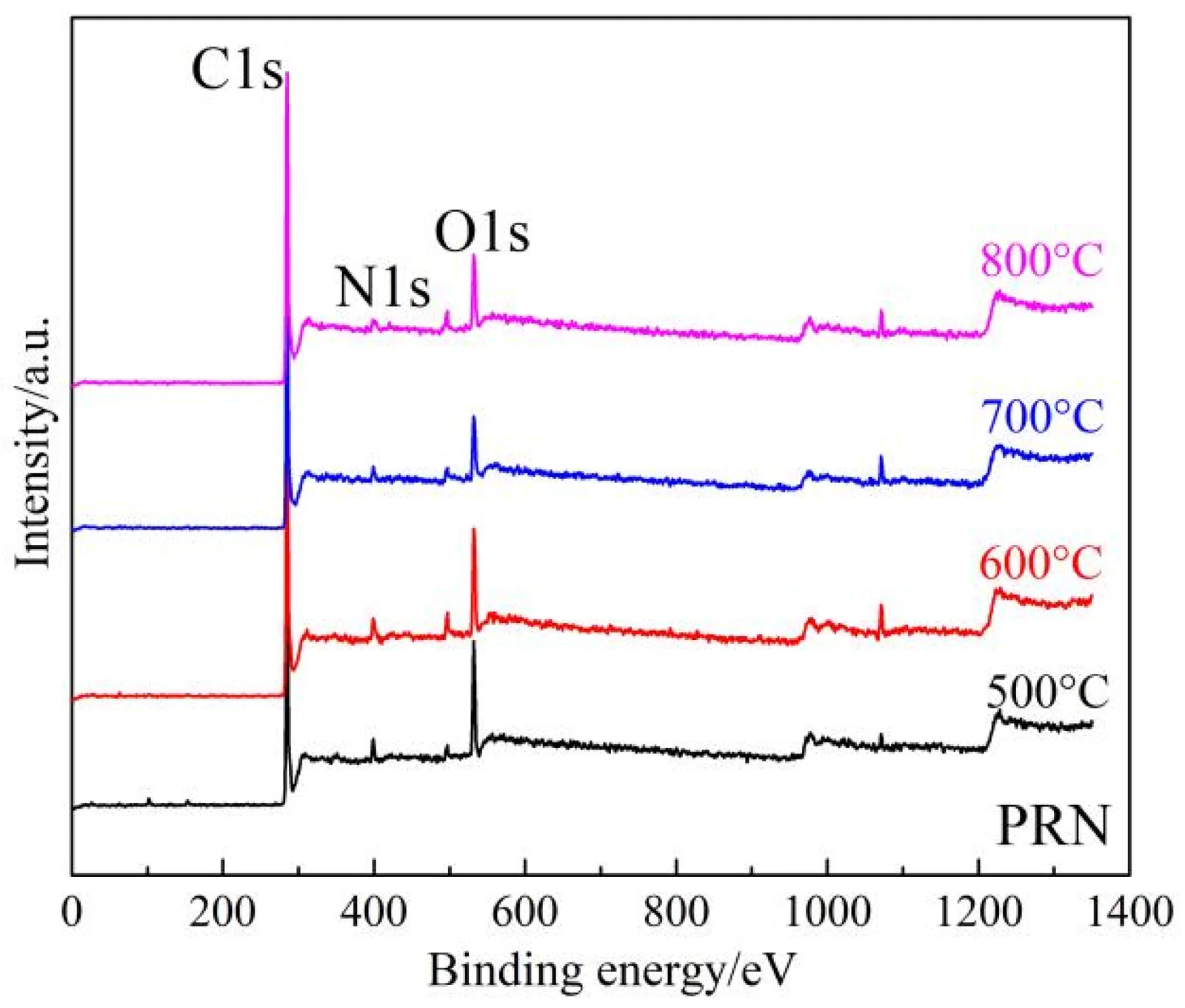
XPS survey spectra of NC samples obtained by calcining PRN at 500–800 °C.

Overall, the FT-IR results indicate that nitrogen introduced by melamine is not present exclusively as a crystalline g-C_3_N_4_ phase and that C–N- and C=N-containing structures persist in the carbonaceous matrix. Because FT-IR cannot unambiguously distinguish nitrogen incorporated into the carbon skeleton from nitrogen in residual carbon nitride, high-resolution XPS analysis was used to determine the dominant nitrogen chemical environments. Figure 7 presents the XPS survey spectra of the NC samples carbonized at 500–800 °C. The characteristic signals centered at approximately 284.8, 398.9, and 532.2 eV are assigned to C 1s, N 1s, and O 1s, respectively [30]. The corresponding surface atomic concentrations calculated from the XPS survey spectra are summarized in Table 1.

The presence of the N 1s signal in all samples confirms that nitrogen introduced by melamine is retained in the phenolic resin pyrolytic carbon over the entire investigated temperature range. Notably, the N 1s peak remains detectable even for NC-800, although the characteristic diffraction peak of g-C_3_N_4_ is no longer clearly observed in the corresponding XRD pattern. This result indicates that the disappearance of crystalline g-C_3_N_4_ at 800 °C does not imply the complete removal of nitrogen; instead, part of the nitrogen may remain chemically incorporated into the carbon skeleton in other bonding configurations. The detailed atomic percentages of C, N, and O are summarized in Table 1, showing that the nitrogen content decreases progressively from 4.71 at% at 500 °C to 1.58 at% at 800 °C, while the carbon content increases from 80.64 at% to 89.91 at%.

Notably, the O 1s peak intensity, as reflected by the oxygen content in Table 1, exhibits a decreasing trend from 600 °C to 800 °C, with oxygen contents of 10.98 at%, 8.05 at%, and 8.51 at% for NC-600, NC-700, and NC-800, respectively. This decrease can be attributed to the progressive thermal decomposition of oxygen-containing functional groups—including phenolic hydroxyl groups (–OH), ether bridges (C–O–C), carbonyl groups (C=O), and carboxyl groups (–COOH)—at elevated temperatures, which release CO, CO_2_, and H_2_O as gaseous products. The elimination of these oxygen-containing species is consistent with the increasing carbon content and the progressive condensation and aromatization of the carbon framework at higher carbonization temperatures. The slight increase in oxygen content from 8.05 at% at 700 °C to 8.51 at% at 800 °C may be attributed to the re-adsorption of oxygen species on the highly porous carbon surface (NC-800 has a specific surface area of 218.9 m^2^/g) during exposure to ambient atmosphere or to the exposure of new carbon surfaces with dangling bonds upon complete decomposition of g-C_3_N_4_ at 800 °C. Nevertheless, the overall trend confirms that higher carbonization temperatures yield a more carbon-rich surface with fewer oxygen-containing functional groups.

**Figure 8:**
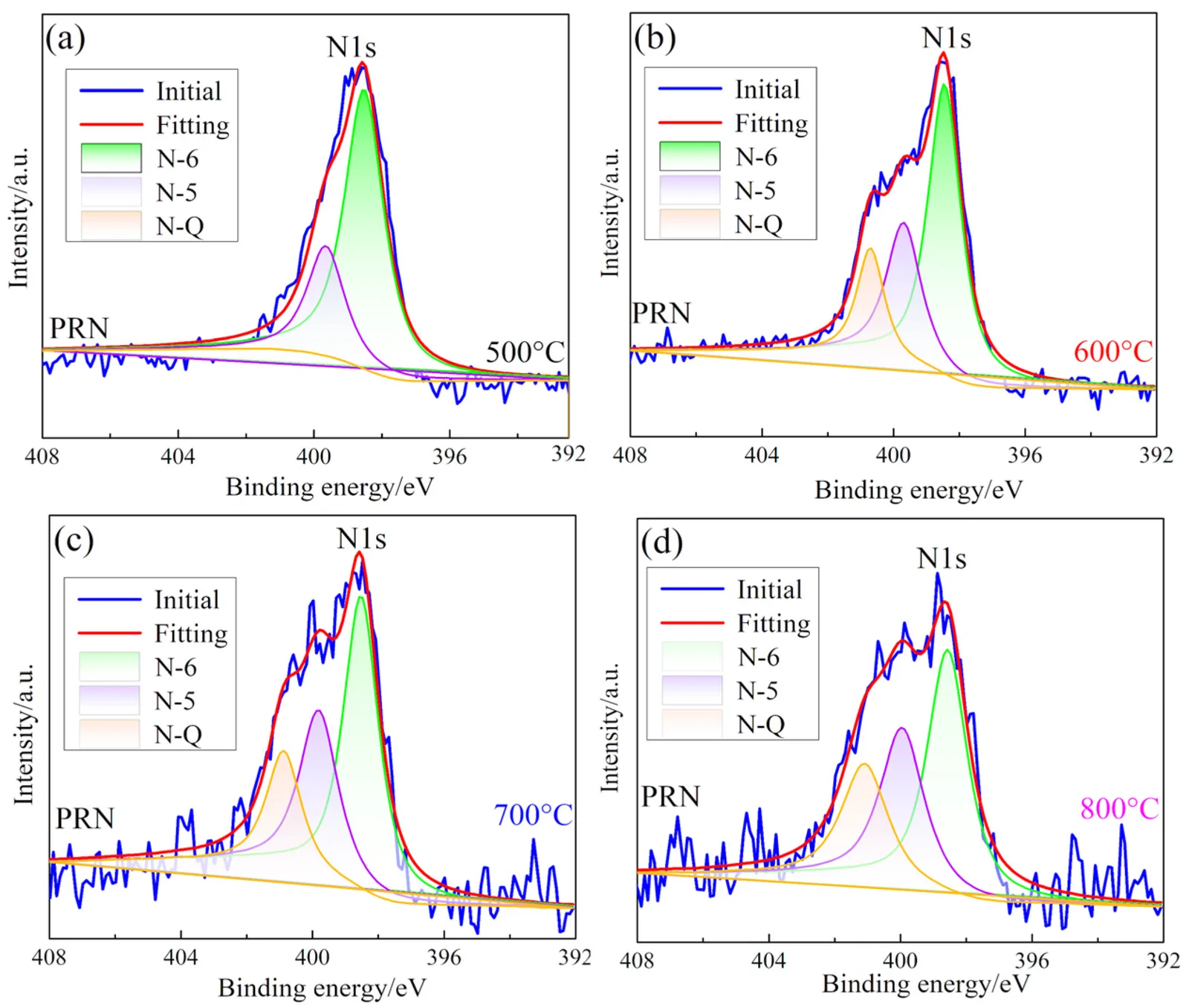
High-resolution N 1s XPS spectra and peak-fitting results of NC samples calcined at different temperatures: (**a**) NC-500, (**b**) NC-600, (**c**) NC-700, and (**d**) NC-800.

To further clarify the chemical states of nitrogen, the high-resolution N1s spectra were deconvoluted, as shown in Figure 8. The N 1s envelope of each sample can be fitted into three components centered at approximately 398.5, 399.6–399.9, and 400.7–401.2 eV, which are assigned to pyridinic nitrogen (N-6), pyrrolic nitrogen (N-5), and graphitic or quaternary nitrogen (N-Q), respectively [31]. These results demonstrate that nitrogen introduced by melamine is incorporated into the phenolic resin pyrolytic carbon skeleton in several structurally modified forms rather than existing exclusively as a separate g-C_3_N_4_ phase. The peak-fitting parameters and relative contents of nitrogen configurations in NC samples calcined at different temperatures are summarized in Table 2. For NC-500, N-6 is dominant, accounting for 72.33% of the fitted N 1s area, while N-5 and N-Q account for 27.20% and 0.47%, respectively. With increasing carbonization temperature, the relative fraction of N-6 decreases continuously from 72.33% at 500 °C to 44.38% at 800 °C, whereas N-Q increases from 0.47% to 24.09%. The N-5 fraction changes comparatively little and remains at approximately 27–32%. The progressive decrease in N-6 and increase in N-Q indicate a temperature-induced transformation of nitrogen configurations. Pyridinic nitrogen is mainly located at the edges or defect sites of aromatic carbon domains and is therefore relatively less thermally stable [32]. As the calcination temperature increases, part of the edge-type nitrogen species may be removed or rearranged, while the remaining nitrogen becomes increasingly incorporated into more stable graphitic positions within the carbon framework. Consequently, the nitrogen structure evolves from edge-dominated configurations toward more thermally stable graphitic nitrogen [33].

This transformation is important for understanding the structural role of nitrogen in the NC samples. Although the total nitrogen content decreases with increasing calcination temperature, the enrichment in the more stable N-Q configuration suggests that a fraction of nitrogen remains firmly embedded in the carbon skeleton. Such nitrogen configurations may modify the local bonding environment and passivate defect-related reactive sites in the glassy carbon matrix. Therefore, the XPS results provide chemical evidence that nitrogen modification affects not only the phase composition but also the defect chemistry of phenolic resin pyrolytic carbon, which may contribute to the subsequent improvement in oxidation resistance.

### 3.3. Pore Structure Evolution of the NC

**Figure 9:**
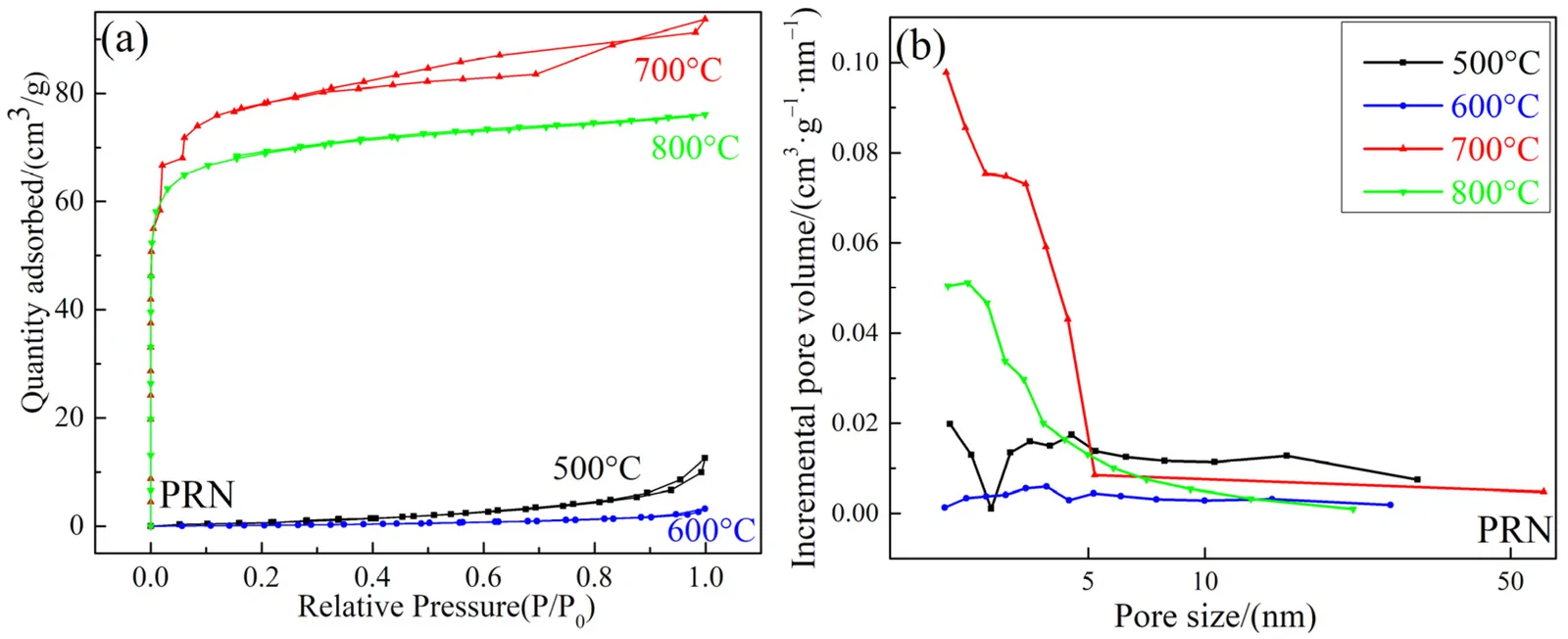
Pore-structure characteristics of NC samples calcined at 500–800 °C: (**a**) N_2_ adsorption desorption isotherms and (**b**) pore-size distributions.

To further clarify the influence of melamine derived nitrogen species on the pore structure evolution of phenolic resin pyrolytic carbon, N_2_ adsorption–desorption measurements were performed on the NC samples calcined at 500–800 °C. The corresponding adsorption–desorption isotherms and pore-size distributions are shown in Figure 9, and the detailed pore-structure parameters are summarized in Table 3.

As shown in Figure 9a and Table 3, NC-500 and NC-600 exhibit relatively low N_2_ uptake, with BET specific surface areas of only 3.6 m^2^/g and 1.1 m^2^/g, respectively. Their total pore volumes are 0.019 and 0.005 cm^3^/g, with negligible microporosity, and their pore structures are dominated entirely by mesopores. In combination with the SEM and TEM observations (Figure 3 and Figure 4), this low uptake can be attributed to the partial filling or blocking of pyrolysis-generated pores by the sheet-like carbon-nitride-rich phase (g-C_3_N_4_). The progressive increase in the crystallinity and stacking order of g-C_3_N_4_ from 500 °C to 600 °C (as evidenced by the sharpening of the (002) diffraction peak in XRD, Figure 2) may further enhance this pore-blocking effect, explaining the even lower surface area of NC-600. In striking contrast, NC-700 shows a dramatic increase in BET specific surface area to 253.6 m^2^/g—an increase of more than two orders of magnitude compared to NC-600—with a total pore volume of 0.145 cm^3^/g and an average pore diameter of only 2.285 nm. The micropore volume of NC-700 reaches 0.098 cm^3^/g, accounting for approximately 67.6% of the total pore volume, while the mesopore volume is 0.047 cm^3^/g. This sample exhibits a pronounced adsorption uptake at low relative pressure (P/P_0_ < 0.01), together with a strong contribution from pores below approximately 5 nm in Figure 9b, indicating the development of abundant micropores and small mesopores [34].

The dramatic pore-structure transformation at 700 °C can be attributed to three synergistic factors supported by complementary characterization evidence. First, the XRD pattern of NC-700 (Figure 2) shows a significantly weakened g-C_3_N_4_ (002) diffraction peak compared to NC-600, indicating substantial decomposition of the crystalline carbon-nitride phase. Second, the XPS N 1s analysis (Figure 7 and Table 2) reveals a marked decrease in the total nitrogen content from 4.12 at% at 600 °C to 3.03 at% at 700 °C, confirming the loss of nitrogen-containing species. Third, the FT-IR spectrum of NC-700 (Figure 5) shows a noticeable weakening of the N–H stretching band (~3000–3600 cm^−1^) and the s-triazine ring breathing mode (~808 cm^−1^), providing further evidence for the decomposition of g-C_3_N_4_ and other nitrogen-containing structures. The decomposition of these phases releases volatile gases (NH_3_, HCN, and N_2_) [35], which escape from the carbon matrix and create abundant micropores. Additionally, the phenolic resin pyrolysis enters its second stage at this temperature, generating additional porosity. The concurrence of these three lines of evidence—XRD, XPS, and FT-IR—strongly supports the proposed mechanism that the decomposition of g-C_3_N_4_ and other nitrogen-containing species is responsible for the dramatic increase in microporosity at 700 °C. For NC-800, a considerable amount of microporosity is still retained (S_BET_ = 218.9 m^2^/g; Vt = 0.072 cm^3^/g), although its nitrogen adsorption capacity is lower than that of NC-700. The micropore volume accounts for approximately 61.0% of the total pore volume, while the mesopore volume remains at 0.046 cm^3^/g. The slight decrease in surface area and pore volume from NC-700 to NC-800 can be attributed to further carbonization and structural contraction, during which some of the smallest pores shrink, merge, or partially collapse. This is consistent with the complete disappearance of the g-C_3_N_4_ diffraction peak in the XRD pattern of NC-800 (Figure 2) and the further reduction in nitrogen content to 1.58 at% (Table 1), indicating that the decomposition of the carbon-nitride phase is essentially complete.

### 3.4. Oxidation Resistance and Structure–Performance Relationship of NC

**Figure 10:**
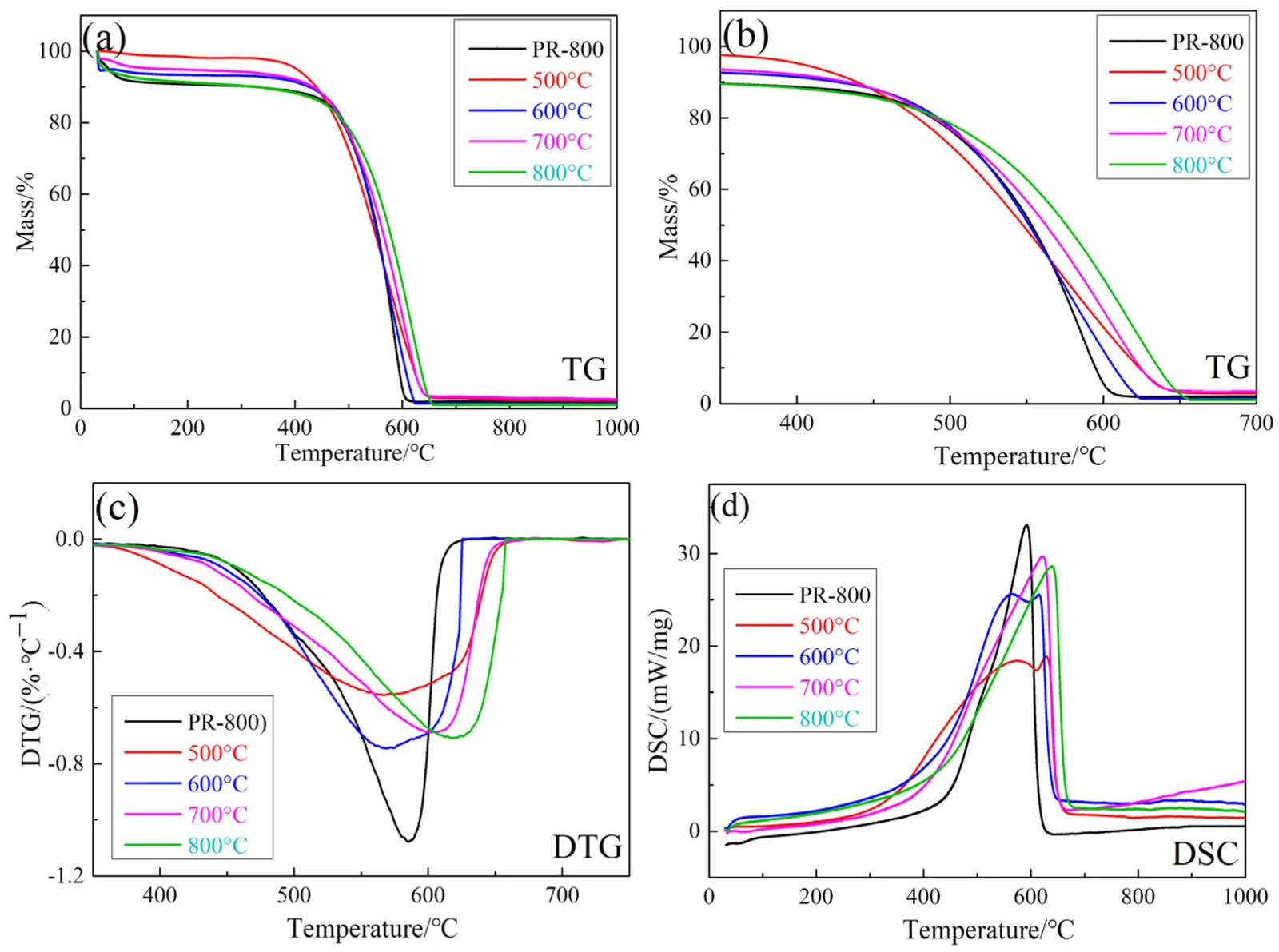
Oxidation behavior of PR-800 and NC-800 °C in air: (**a**) TG curves over the full temperature range, (**b**) enlarged TG curves in the main mass-loss region, (**c**) DTG curves, and (**d**) DSC curves.

The oxidation resistance of PR-800 and NC-800 was evaluated by TG-DTG-DSC analysis in air, as shown in Figure 10. Because both samples were carbonized at 800 °C under identical conditions, their comparison directly reflects the influence of melamine-derived nitrogen modification. The TG curves in Figure 10a,b show that NC-800 retains a higher mass fraction during the main oxidation stage, and its rapid mass-loss region shifts toward higher temperatures relative to PR-800. This result indicates that nitrogen modification delays the oxidation of phenolic resin pyrolytic carbon. The DTG curve of NC-800 exhibits a lower absolute maximum mass-loss rate and a peak shifted to higher temperature compared with PR-800 (Figure 10c), demonstrating that the oxidation reaction proceeds more slowly and over a broader temperature interval. Consistently, the DSC exothermic peak of NC-800 is broader, lower in intensity, and located at a higher temperature than that of PR-800 (Figure 10d). These features indicate that nitrogen modification suppresses rapid carbon consumption and delays the intensive heat-release stage associated with oxidation. The enhanced oxidation resistance of NC-800 may arise from the combined effects of nitrogen incorporation and pore-structure regulation. Nitrogen retained as pyridinic-N, pyrrolic-N, and particularly thermally stable graphitic-N may modify the local bonding environment and reduce the reactivity of oxidation-active carbon sites. Meanwhile, the modified pore structure may increase the resistance to oxygen transport within the carbon matrix. Overall, the rightward shifts in the TG, DTG, and DSC features, together with the reduced mass-loss and heat-release rates, confirm that nitrogen modification effectively improves the oxidation resistance of phenolic resin pyrolytic carbon.

**Figure 11:**
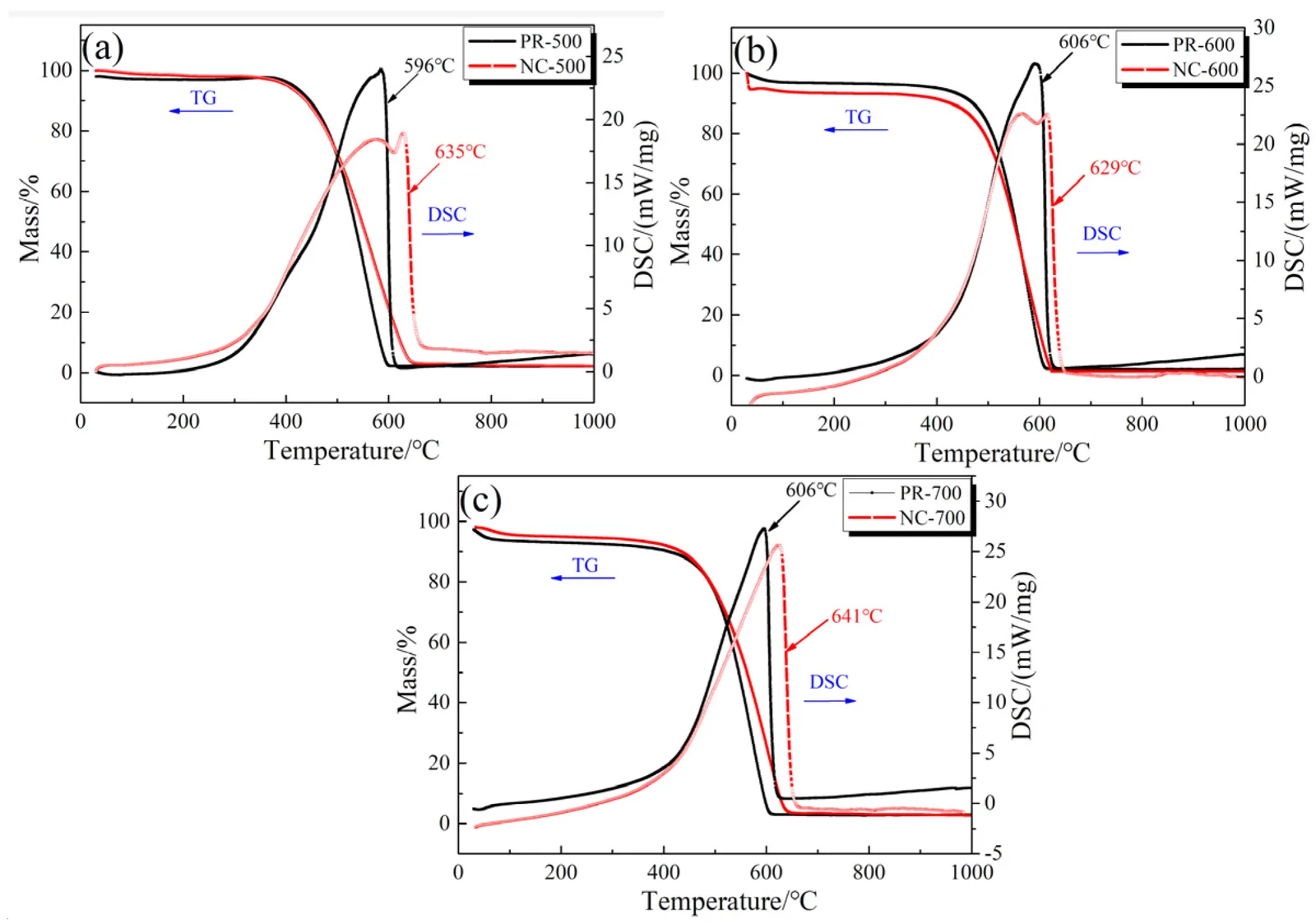
TG–DSC curves in air of PR and NC carbonized at different temperatures: (**a**) PR-500 and NC-500, (**b**) PR-600 and NC-600, and (**c**) PR-700 and NC-700.

To establish a comprehensive correlation between nitrogen content, g-C_3_N_4_ phase evolution, and oxidation resistance, TG-DSC analysis was performed on all NC samples (NC-500 to NC-800) as well as the corresponding unmodified PR samples (PR-500 to PR-800). TG–DSC measurements were performed on the paired PR and NC samples carbonized at 500, 600, and 700 °C, as shown in Figure 11.

The results demonstrate a consistent delay in the final oxidation stage after nitrogen modification. For the samples carbonized at 500 °C, the completion temperature of the main oxidation process increases from approximately 596 °C for PR-500 to 635 °C for NC-500, corresponding to an improvement of approximately 39 °C (Figure 11a). At a carbonization temperature of 600 °C, the corresponding temperature increases from 606 °C for PR-600 to 629 °C for NC-600, giving an increase of approximately 23 °C (Figure 11b). A more pronounced improvement is again observed at 700 °C, where the complete oxidation temperature increases from approximately 606 °C for PR-700 to 641 °C for NC-700, corresponding to an increase of approximately 35 °C (Figure 11c). The DSC curves further show that the exothermic oxidation process of the NC samples generally extends over a broader high-temperature range, while the maximum exothermic response is reduced relative to the corresponding PR samples. These features indicate that nitrogen modification suppresses rapid carbon consumption and delays the completion of the oxidation process.

When the results in Figure 11 are considered together with those of PR-800 and NC-800 in Figure 10, nitrogen modification is found to improve the oxidation resistance over the entire investigated carbonization-temperature range of 500–800 °C. Importantly, the magnitude of the improvement is not strictly monotonic with carbonization temperature: the increases in complete oxidation temperature are approximately 39, 23, 35, and 40 °C for the samples carbonized at 500, 600, 700, and 800 °C, respectively. This non-monotonic behavior indicates that oxidation resistance is governed by the coupled evolution of phase composition, pore accessibility, and nitrogen configuration rather than by the amount of a single nitrogen-containing phase. At 500–700 °C, the XRD, SEM, and TEM results reveal the presence of sheet-like carbon-nitride-rich structures consistent with g-C_3_N_4_. These structures are distributed on the carbon surface and partially occupy the pore space and may therefore increase the resistance to oxygen transport into the carbon matrix. This interpretation is also consistent with the relatively low accessible surface area of NC-500 and NC-600. As the carbonization temperature increases toward 700 °C, the carbon-nitride-rich phase progressively decreases, while partial decomposition and structural rearrangement are accompanied by the development of abundant micropores and small mesopores. The resulting increase in oxygen-accessible surface area may partially counteract the protective effect associated with pore blocking, which may account for the non-monotonic variation in the degree of oxidation improvement. At 800 °C, the characteristic XRD peak of crystalline g-C_3_N_4_ is no longer clearly detected; nevertheless, Figure 9 shows that NC-800 still exhibits an approximately 30 °C increase in onset oxidation temperature and an approximately 40 °C increase in complete oxidation temperature relative to PR-800. In addition, the lower maximum mass-loss rate in the DTG curve and the broader and delayed DSC exothermic response of NC-800 demonstrate that the oxidation reaction proceeds more slowly. XPS provides an important explanation for this behavior: although the total nitrogen content decreases with increasing carbonization temperature, nitrogen remains incorporated into the carbon framework, and the relative fraction of thermally stable graphitic-N increases substantially. Therefore, the oxidation-resistance enhancement at high carbonization temperature cannot be attributed solely to an intact g-C_3_N_4_ protective phase. Instead, the results support a temperature-dependent dual-function mechanism in which physical regulation of oxygen-access pathways by the carbon-nitride-rich phase contributes at intermediate temperatures, while chemical regulation of oxidation-active carbon sites by skeletal nitrogen becomes increasingly important as the carbonization temperature increases.

**Figure 12:**
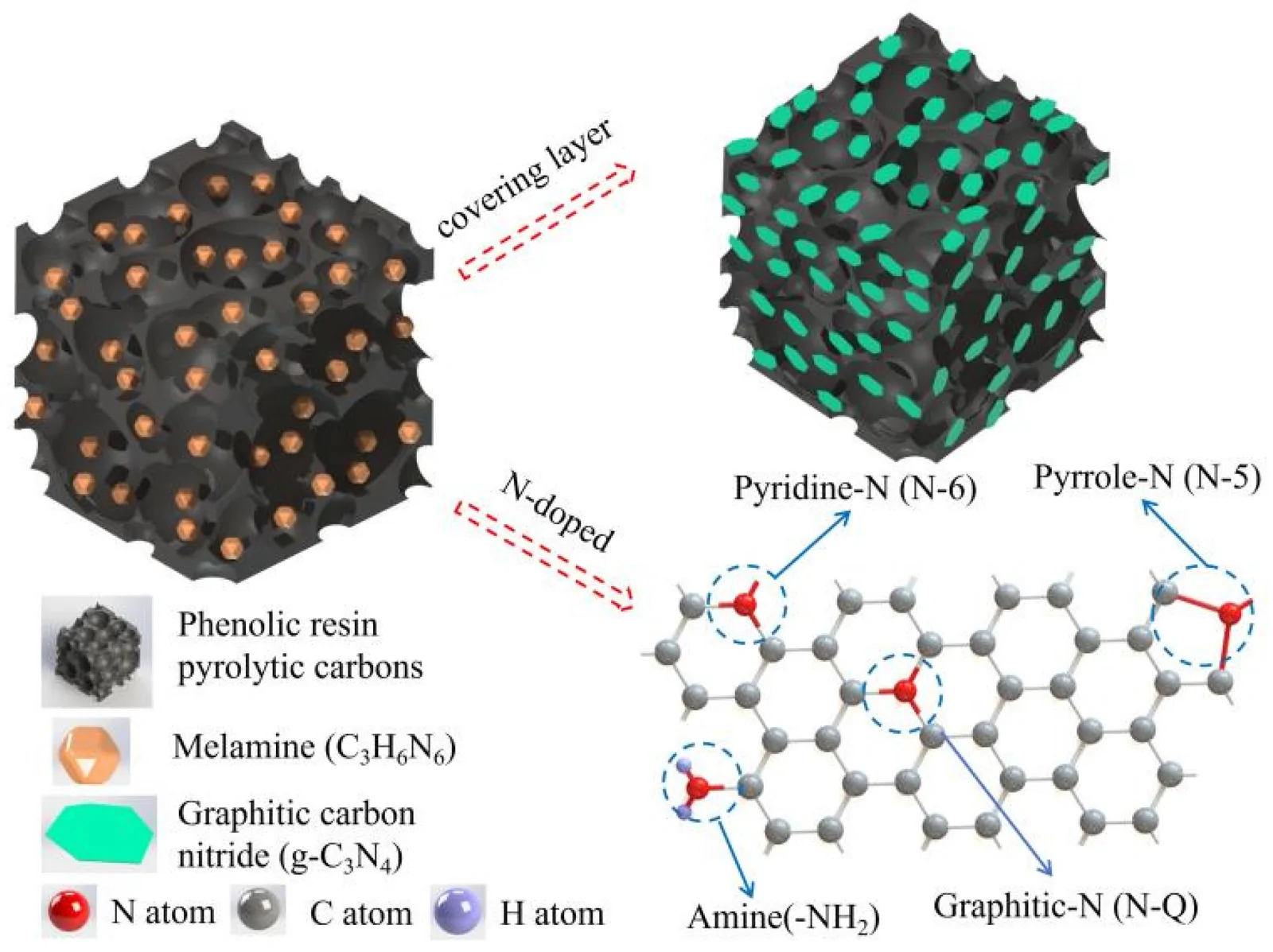
Schematic illustration of the effect of melamine introduction on the structure evolution of phenolic resin pyrolytic carbon.

Figure 12 schematically illustrates the structural evolution of phenolic resin pyrolytic carbon after melamine introduction. During pyrolysis, melamine affects the carbon structure through two main pathways. On the one hand, part of the melamine-derived species forms sheet-like graphitic carbon nitride (g-C_3_N_4_), which is distributed on the pore walls or within the pore channels of the resin-derived carbon matrix, acting as a partial covering or filling phase. On the other hand, part of the nitrogen is chemically incorporated into the carbon skeleton in the form of pyridinic-N (N-6), pyrrolic-N (N-5), and graphitic-N (N-Q).

These two structural effects may jointly influence the oxidation behavior of the material. The g-C_3_N_4_ covering layer may partially block open pores and hinder oxygen diffusion into the carbon matrix, whereas the incorporation of nitrogen into the carbon skeleton may modify the local bonding environment and passivate oxidation-active defect sites. Therefore, melamine introduction not only changes the pore structure of phenolic resin pyrolytic carbon but also regulates the chemical states of nitrogen in the carbon framework, which together may contribute to the improved oxidation resistance of the NC samples.

## 4. Conclusions

In this work, nitrogen-modified phenolic resin pyrolytic carbons (NC) were successfully prepared by introducing melamine into phenolic resin prior to carbonization at 500–800 °C. The forms of nitrogen, the evolution of pore structure, and the oxidation resistance of the resulting carbons were systematically investigated. The main conclusions are summarized as follows.

(1) Melamine-derived nitrogen is retained in two principal forms. XRD, SEM, and TEM indicate that a sheet-like carbon-nitride-rich phase consistent with g-C_3_N_4_ forms in NC-500 to NC-700 and progressively decreases with increasing carbonization temperature. At 800 °C, long-range crystalline g-C_3_N_4_ is no longer detected. FT-IR and XPS show that nitrogen also remains in chemically bonded pyridinic-N, pyrrolic-N, and graphitic-N configurations within the carbonaceous framework.
(2) The phase composition, nitrogen configurations, and pore structure evolved markedly with carbonization temperature. The g-C_3_N_4_ phase was retained mainly at 500–700 °C and gradually decreased at higher temperatures, whereas the proportion of thermally stable graphitic-N increased. At the same time, the pore structure changed from predominantly mesoporous at 500–600 °C to micropore-rich at 700–800 °C because of the competing effects of pore filling, decomposition-induced pore formation, and structural contraction.
(3) Compared with unmodified PR-800, NC-800 exhibited an increase of approximately 30 °C in the onset oxidation temperature and an extension of about 40 °C in the complete oxidation temperature. Its lower maximum oxidation rate and delayed exothermic response further confirmed the improved oxidation resistance. This enhancement is attributed to the synergistic effects of physical shielding by melamine-derived g-C_3_N_4_ and chemical regulation of oxidation-active carbon sites by incorporated nitrogen. These findings demonstrate that melamine modification is a simple and potentially scalable approach for improving the oxidation resistance and extending the high-temperature service life of phenolic-resin-based carbon materials.

## Figures and Tables

**Table 1:** Surface atomic concentrations of C, O, and N in NC calcined at different temperatures determined by XPS survey spectra.

Sample	C (%)	O (%)	N (%)
NC-500	80.64	14.65	4.71
NC-600	84.90	10.98	4.12
NC-700	88.92	8.05	3.03
NC-800	89.91	8.51	1.58

**Table 2:** Peak-fitting parameters and relative contents of nitrogen configurations in NC samples calcined at different temperatures.

Sample	N-6 (Pyridine-N)			N-5 (Pyrrolic-N)			N-Q (Graphitic-N)		
	Position	Area	Content	Position	Area	Content	Position	Area	Content
NC-500	398.5	2290	72.33%	399.6	861	27.20%	401.2	15	0.47%
NC-600	398.5	2320	54.85%	399.7	1203	28.45%	400.7	706	16.70%
NC-700	398.5	1134	49.74%	399.8	708	31.05%	400.9	438	19.21%
NC-800	398.6	942	44.38%	399.9	669	31.53%	401.1	511	24.09%

**Table 3:** Pore structure parameters of NC samples calcined at different temperatures.

Sample	S_BET_/(m^2^·g^−1^)	V_t_/(cm^3^·g^−1^)	D_p_/(nm)
NC-500	3.624	0.019	21.400
NC-600	1.104	0.005	17.782
NC-700	253.566	0.145	2.285
NC-800	218.893	0.118	2.997

## Data Availability

The original contributions presented in this study are included in the article. Further inquiries can be directed to the corresponding authors.
